# Fear of Falling, Fall Risk, Depression, and Anxiety in Community-Dwelling Older Adults

**DOI:** 10.1093/geroni/igab046.3697

**Published:** 2021-12-17

**Authors:** Aleatha Rossler, Ladda Thiamwong, Rui Xie, Jeffrey Stout, Joon-Hyuk Park, Renoa Choudhury, Oscar Garcia

**Affiliations:** 1 University of Central Florida, Orlando, Florida, United States; 2 College of Nursing, University of Central Florida, Orlando, Florida, United States

## Abstract

Fear of falling is common in the older adult population with an estimated 43% being affected. We aimed to examine the associations among fear of falling, fall risk, depression, and anxiety in community-dwelling older adults. For this study 124 participants ranging from 60 to 96 years of age were recruited from the community settings in Central Florida. Fear of falling, fall risk, depression and anxiety were assessed using the Falls Efficacy Scale International (FES-I), the CDCSTEADI fall risk assessment, the Patient Health Questionnaire (PHQ) for depression, and the Geriatric Anxiety Inventory Short form (GAI-SF) for anxiety respectively. Data was collected via the Qualtrics survey. Comparisons were made for those below age 75 and those aged 75 and older, with 51.6% being under 75. Four ethnicity categories were also used: African American (8.1%), Asian (2.4%), Hispanic (14.5%), and non-Hispanic white (75%). All participants scored above 4 on the STEADI scale indicating fall risk. 42 scored positive for fear of falling on the FES-I scale and of that 42, 35.7% had a history of one or more falls in the last year (p < .01). 46.8% of the participants screened positive for depression and 100% of participants were positive for anxiety. Using one-way ANOVA analysis, we found significant relationships between (1) depression (p<.01); (2) STEADI (p<.01) and FES-I.

